# Correction: How fake news can turn against its spreader

**DOI:** 10.1371/journal.pone.0339315

**Published:** 2025-12-19

**Authors:** The PLOS One Staff

## Notice of Republication

This article was republished on December 5, 2025, to correct an error in which several blocks of text were unintentionally deleted from the manuscript. Specifically, text was missing from pages 2 through 6, pages 9 and 10, and page 13.

The publisher apologizes for these errors. The originally published, uncorrected article and the republished, corrected articles are provided here for reference.

## Supporting information

S1 File Originally published, uncorrected article. (PDF)

S2 File Republished, corrected article. (PDF)
