# Supplementary material for: How fake news can turn against its spreader
Source: PLoS One. 2025 Sep 5;20(9):e0331608. doi: 10.1371/journal.pone.0331608 (PMC12413092; doi:10.1371/journal.pone.0331608)
Supplement: Attachment — Submitted filename: Response to Reviewers.pdf [file pone.0331608.s003.pdf]

TOKYO METROPOLITAN UNIVERSITY

Faculty of Economics and Business Administration
Graduate School of Management

18F Marunouchi Eiraku Building, 1-4-1 Marunouchi,
Chiyoda-ku, Tokyo 100-0005, Japan

Tomooki Yuasa
Associate Professor

T: +81-3-6268-0521
F: +81-3-6268-0522

tomooki-yuasa@tmu.ac.jp
https://www.tmu.ac.jp/stafflist/data/ya/31719.html

12 August 2025

Dear Editor,

How fake news can turn against its spreader (PONE-D-25-21796)

Thank you for your letter of 11 June and the attached referee reports. We found both referee comments helpful to improve the presentation and attraction of our paper, and we have revised our paper accordingly, taking into account all the points raised by the referees. Response to specific points raised by the referees, and changes made to the paper, are attached below. To make it easier to identify changes made to the paper, we have marked them in blue. We hope the revised manuscript is now suitable for publication in PLOS One.

Sincerely yours,

Tomooki Yuasa

Response to Referee 1

We thank the referee for their suggestion of enhancing the numerical experiments in the paper. We agree that such an analysis will improve the quality of the paper, and accordingly we have carried out further numerical analysis. Specifically, the single sample of Figure 1 in the original manuscript has been sampled ten thousand times to take the average to show the statistics of the effect identified in our paper. These are shown as Figure 2 in the revised manuscript. The simulated sample paths in Figure 2 of the original manuscript have been expanded with different parameter values, shown in Figure 3 of the revised manuscript. Further, to show the corresponding statistics, ten thousand simulations have been carried out to show the averaged effect, included in Figure 4 of the revised manuscript.

Response to Referee 2

We thank the referee for making a number of interesting and valuable suggestions, all of which we agree would enhance the interest of our paper. Our response to the specific points raised by the referee are as follows.

1. Positioning in relation to prior work. We thank the referee for pointing out these interesting papers that examine belief polarisation effect using Bayesian net and other approaches. In the revised manuscript we have briefly commented on these papers, in the paragraph starting at the bottom of page 3 of the revised manuscript.
2. Clarification of model assumptions. We agree with the referee that, while we have stated the assumptions in our original submission, further remarks on these assumptions would be highly beneficial. In the revised manuscript we have inserted a paragraph commenting on the model parameter conditions, in the paragraph starting towards the bottom of page 6 of the revised manuscript.
3. Consideration of human cognitive constraints. We agree this is an important (and can also be contentious) issue that should be discussed at least briefly. In the revised manuscript we have included a brief discussion on the issue of the use of Bayesian method in our paper, in the paragraph starting at the bottom of page 3. One important point here is that a number of well-documented violations of the Bayesian updating can be explained based on a framework that generalised the Bayes formula, and our approach remains applicable in the broader framework. We have commented briefly on this, although we did not elaborate on it because it is tangential to the effect investigated in the present paper.
4. Practical and policy implications. We agree with the referee that such a discussion would increase the paper's relevance. In the revised manuscript we have inserted a discussion on this, in the penultimate paragraph of the paper.
